# Reply to Ståhl et al. Comment on “Honndorf et al. Comparative Analytics and Pharmacodynamics of the Complex Protein-Free Botulinum Toxin Type A Formulations DaxibotulinumtoxinA, IncobotulinumtoxinA and RelabotulinumtoxinA. *Toxins* 2026, *18*, 142”

**DOI:** 10.3390/toxins18070292

**Published:** 2026-07-03

**Authors:** Stefanie Honndorf, Katja Kühbach, Karl-Heinz Eisele, Alina Shokurova, Philipp Buch, Claudia Jatzke, Harold Victor Taylor, Klaus Fink

**Affiliations:** Merz Therapeutics GmbH, Neurotoxin & Biotechnology Development, Eckenheimer Landstr. 100, 60318 Frankfurt, Germany

We appreciate the comment [1] on our recent paper in which three botulinum toxin type A products were compared. All products are 150 kDa toxins, i.e., purified from the complexing proteins, and are approved in several countries. The study shows that there are still subtle differences. The paper publishes a preclinical study and does not contain clinical data or clinical conclusions. These preclinical paradigms are, in our opinion, simpler and, therefore, more straight forward than a clinical comparison and make, with all limitations, these comparisons of the formulations possible. In contrast to clinical data, each preclinical test system focuses on few or just one aspect, e.g., neurotoxin protein content, biological activity, or paralysis in the mouse Digit Abduction Score assay. The totality of data allows the reader to understand and assess differences between the compared products.

We observed membrane permeabilization after testing the membrane integrity and a reduced viability in the neuronal cell cultures used for BoNT/A potency measurement when samples containing 1.1 mg/mL polysorbate 80 (PS80) were analyzed. These cytotoxic effects can be reproduced by any lab using the cell culture techniques described in our paper. Other researchers have found similar results using different cell types; cellular stress response was increased in human dermal fibroblasts, human epidermal keratinocytes and human umbilical vein endothelial cells within 4 h of exposure to PS20 or PS80 at 1 mg/mL [2]. In another study, it was shown that incubation of human lymphoma cells, as well as human adult low-calcium temperature keratinocytes with increasing concentrations of PS80, led to cytotoxic effects after 1 h, revealing a half-maximal cytotoxic concentration (CC50) of 0.3 mg/mL for both cell types [3]. Cytotoxicity results from the amphiphilic, i.e., surface-active, character of polysorbates [4]. The polyoxyethylene chains are hydrophilic while the oleic acid fatty chain is hydrophobic, which, depending on concentration and duration of exposure, can disturb the structure and function of cell membranes. While this is well-known, it is not an artifact but in fact the basic characteristic of a detergent such as PS80 [5]. If intramuscularly injected into a patient, the PS80-containing formulation may become diluted because the muscle is perfused and other clinical factors may be of influence as well. However, it was not the objective of the study to mimic a clinical setting. Detergents such as PS80 are frequently added to proteinaceous formulations as a stabilizer to avoid denaturation and aggregation. According to the FDA Inactive Ingredients Database of April 2026 [6], PS80 or polyoxyethylene (80) sorbitan monooleate may be used as an excipient in intramuscular injection solutions at concentrations up to 5% (*w*/*v*). This does not change the fact that it is a detergent and that detergents interact and destabilize membranes. In addition, polysorbates are susceptible to oxidative degradation, releasing peroxides and fatty acids [7,8] which have been demonstrated to adversely affect the stability of biopharmaceuticals.

The study demonstrated that INCO and RELA have similar relative biological activity and neurotoxin protein content, resulting in similar specific activity (266.68 ± 19.97 and 252.89 ± 7.62 U/ng, respectively). Since the biologic activity and protein content were determined by the same assays, we think that the resulting figures can be compared in this system, irrespective of the general non-comparability of manufacturer units. Contrary to what the comment claims, it was concluded in the paper that the comparable bioactivity of INCO and RELA in the in vitro assays supports the published clinical experience with both products [9,10]. The conclusion is therefore that 20 U INCO and 20 U RELA should have the same effect on patients as 50 U INCO and 50 U RELA. Since RELA was studied clinically only at 50 U for the treatment of glabellar frown lines, a unique study with INCO using this dose was the study by Kerscher [9], which did show similar clinical effects. There was, however, no implicit suggestion to administer unapproved higher doses of INCO because it would not make sense in light of the biochemical similarity of INCO and RELA and the nonclinical nature of the data. That being said, we did not see a good reason from the in vitro assays to sacrifice animals (digit abduction scoring, DAS) to study the potency and duration of the effects of RELA in vivo. The sentence in the abstract that INCO, DAXI and RELA were compared in vitro and in vivo refers to the DAS performed on INCO and DAXI only. The maximum dose of up to 40 U/kg used in the DAS has been used before [11,12] and caused a submaximal local paralysis (mean peak DAS of 3.8 for INCO and 3.9 for DAXI; max. score would be 4.0) and all animals completed the study in good health. It is well-known that BoNT/A injections cause a transient reduction in body-weight increase [12,13], which was observed in our study as well. Taken together, the dose of 40 U/kg is scientifically sound and the results are reliable.

It is further commented that our calculation of the specific activity (biological activity/neurotoxin protein mass) was incorrectly based on labeled units that are incomparable. That is incorrect because the specific activity was not calculated with the labeled units but with the biological activity measured in our cell-based potency assay and the protein mass measured in our ELISA.

The investigation of spread after intramuscular injection was purely experimental and as such not a validated assay. This is the scientific standard because preclinical determinations and characterizations are mostly performed in non-validated assay systems and are accepted by regulatory agencies. Spread is not a standardized concept in pharmacology, other than diffusion, but it is a frequently used term in BoNT therapy. Spread, in our view, is affected by the injection site, injection speed, injection volume, muscular activity, rubbing/massaging of the injection site and tissue drainage, whereas diffusion is a well-defined physical process adhering to Fick’s laws and which is dependent upon parameters such as molecular weight, concentration gradient, temperature and mixture density. There is no generally accepted or established experimental set-up to study spread according to any definition, but it has been studied using magnetic resonance imaging [14], ultrasound [15], sudometry with iodine starch [16] or staining [17,18], which all are experimental non-validated approaches.

The simple nonclinical methodologies applied in the study are suitable for addressing the scientific questions and are precisely targeted at rectifying deceptive assertions. The conclusions are based on the results and may be reproduced by other laboratories. For this reason, we must reject the allegations of unsupported interpretations and inaccurate conclusions.
